# Ethnic-specific *WRN* mutations in South Asian Werner syndrome patients: potential founder effect in patients with Indian or Pakistani ancestry

**DOI:** 10.1002/mgg3.1

**Published:** 2013-03-28

**Authors:** Bidisha Saha, Davor Lessel, Sheela Nampoothiri, Anuradha S Rao, Fuki M Hisama, Dincy Peter, Chris Bennett, Gudrun Nürnberg, Peter Nürnberg, George M Martin, Christian Kubisch, Junko Oshima

**Affiliations:** 1 Department of Pathology and Division of Medical Genetics, University of WashingtonSeattle, WA; 2 Institute of Human Genetics, University of UlmUlm, Germany; 3 Department of Pediatric Genetics, Amrita Institute of Medical Sciences and Research CenterKochi, Kerala, India; 4 Department of Ophthalmology, Amrita Institute of Medical Sciences and Research CenterKochi, Kerala, India; 5 Department of Dermatology, Venerology and Leprology, Christian Medical CollegeVellore, Tamil Nadu, India; 6 Yorkshire Regional Genetic Service, Chapel Allerton HospitalLeeds, United Kingdom; 7 Cologne Center for Genomics, University of CologneCologne, Germany; 8 Center for Molecular Medicine Cologne, University of CologneCologne, Germany; 9 Cologne Excellence Cluster on Cellular Stress Responses in Aging-Associated Diseases, University of CologneCologne, Germany

**Keywords:** Founder mutations, India, Pakistan, segmental progeroid syndromes, Werner syndrome, *WRN*

## Abstract

Werner syndrome (WS) is a rare autosomal recessive disorder characterized by multiple features consistent with accelerated aging. It is caused by mutations in the *WRN* gene, which encodes a RecQ type helicase. To date, more than 70 disease-causing mutations have been reported. While founder mutations and a corresponding relatively high incidence of WS have been reported in Japan and Sardinia, such mutations have not been previously described among patients of South Asian descent. Here, we report two novel *WRN* mutations in three pedigrees. A homozygous c.561A>G mutation in exon 6 was identified both in a pedigree from Kerala, India and in a British patient of Pakistani ancestry. Although c.561A>G does not alter the corresponding amino acid (p.Lys187), it creates a cryptic splice site resulting in a 98 bp deletion at the mRNA level (r.557_654del98) followed by a frameshift (p.Lys187Trpfs*13). These two cases shared the same haplotype across the *WRN* gene, and were distinct from another Indian Werner patient with a homozygous stop codon mutation, c.2855 C > A (p.Ser952*), in exon 24. As the Indian population increases and the awareness of WS grows, we anticipate that more cases will be identified with these founder mutations among South Asian WS patients.

## Introduction

Werner syndrome (WS) is an adult-onset segmental progeroid syndrome caused by mutations at the *WRN* locus (Yu et al. 1996). Affected individuals undergo relatively normal development until they reach adolescence (Epstein et al. 1966; Martin 1978; Goto 1997). An early feature, often recognized retrospectively, is the lack of an adolescent growth spurt. WS patients begin to develop an aged appearance in their 20s and 30s with the onset of thinning, gray hair, skin atrophy, loss of subcutaneous fat, “bird-like” pinched face, and a hoarse voice. They also develop various age-related disorders at earlier ages compared with the general population. These include bilateral ocular cataracts, type 2 diabetes mellitus, osteoporosis, osteosclerosis, hypogonadism, atherosclerosis, and malignancies (Goto et al. 1996, 1997; Oshima et al. 2012). Among these, ocular cataracts that require surgical intervention in the early 30s are seen in virtually all cases with *WRN* mutations (Huang et al. 2006). An epidemiological study of WS patients in Japan reported that 78% of cases showed soft tissue calcification in the Achilles tendon detected by X-ray (Takemoto et al. 2012). This soft tissue calcification may be responsible for the deep ulcerations in the ankles and elbows that are highly characteristics of WS. The most common causes of death in WS patients are cancer and myocardial infarction with the median age of death being 56 years (Huang et al. 2006).

The *WRN* gene encodes a 180 kD multifunctional nuclear protein (Yu et al. 1996; Bohr et al. 2002) with 3′→5′ exonuclease (amino acid, aa 60–230) (Yu et al. 1996; Gray et al. 1997; Kamath-Loeb et al. 2012) and 3′→5′ helicase (aa 556–878) (Huang et al. 1998) domains. A major nuclear localization signal is located at the C-terminus (Matsumoto et al. 1998). A series of biochemical, cell biological, and animal studies of the WRN protein revealed its critical roles in telomere maintenance. Cells from WS patients undergo accelerated replicative senescence and telomere attrition (Opresko 2008). WRN has been shown to be involved in lagging strand synthesis (Crabbe et al. 2004). In the absence of WRN, sister chromatids undergo accelerated shortening, telomere fusion, and cell death (Crabbe et al. 2007). *WRN* knockout mice do not exhibit an accelerated aging phenotype but do so six generations after being crossed with Terc knockout mice (Chang et al. 2004). More recently, *WRN* was shown to be required for the telomere association of SIRT6, a DNA damage-related histone deacetylase (Michishita et al. 2008). These findings point to a role for *WRN* in telomere maintenance.

We previously reported that the p.Leu1074Phe polymorphism of WRN protein is enriched in Finnish centenarian and Mexican adult populations and that they may alter the age-dependent risk of atherosclerosis (Castro et al. 2000). Recently, a genome-wide association study of centenarians identified the *WRN* p.Leu1074Phe polymorphism as a longevity-associated variant (Sebastiani et al. 2012). p.Leu1074Phe resides in the RecQC domain (aa 949-1092) and overlaps with the region that interacts with substrate DNA and various proteins, including TRF2 (Nora et al. 2010). One possible mechanism of longevity assurance by *WRN* might be to optimize the coordination of interacting proteins and to modulate the helicase and/or exonuclease activities of the WRN protein complex at the targeted sites.

To date, more than 70 different *WRN* mutations have been identified (Matsumoto et al. 1997; Huang et al. 2006; Uhrhammer et al. 2006; Friedrich et al. 2010; Takada-Watanabe et al. 2012). The majority of disease-causing mutations result in either a premature stop codon, a small indel, or splice site mutations that result in the non-sense mediated decay of mutant mRNA, and/or deletion of the nuclear localization signals (Suzuki et al. 2001). A few missense mutations and genomic rearrangements have also been reported (Friedrich et al. 2010). While many of them are private mutations, founder effects have been established at least in two ethnic groups, the Japanese and the Sardinians (Yu et al. 1996; Satoh et al. 1999; Masala et al. 2007; Zlotogora 2007). Here, we report original findings of two novel *WRN* mutations found in three Indian and Pakistani pedigrees together with their clinical phenotypes.

## Materials and Methods

### Ethics statement

Patients signed the approved consent forms to participate in the studies of International Registry of Werner Syndrome (Seattle, WA) prior to the initiation of the study. This study was approved by the Human Subjects Division, Institutional Review Board of the University of Washington, Seattle, WA.

### Patient recruitment

Patients were referred to our International Registry of Werner Syndrome (http://www.wernersyndrome.org) for clinical assessments and molecular diagnosis. Enrollment in the study was primarily based on the clinical interpretations of referring physicians. Given the interest of the Registry in atypical forms of the WS (Chen et al. 2003), we accept patients with somewhat unusual presentations. Revised clinical criteria have been previously described (Oshima et al. 2012). Cardinal signs include bilateral ocular cataracts, characteristic skin findings, short stature, graying and loss of hair, and parental consanguinity. Other signs include type 2 diabetes mellitus, osteoporosis, evidence of premature atherosclerosis, malignances, voice changes, and flat feet.

Details of biological sample processing were described previously (Huang et al. 2006). For this study, blood samples shipped directly from India were used to establish lymphoblastoid cell lines (LCLs). Genomic DNA was isolated from both blood samples and LCLs. mRNA and proteins were isolated from LCLs, as previously described (Huang et al. 2006; Friedrich et al. 2010).

### Nucleotide sequencing and Western blot analysis

The detailed methodologies of genomic polymerase chain reaction (PCR) and reverse transcription PCR (RT-PCR) sequencing were as described previously (Oshima et al. 1996; Huang et al. 2006; Friedrich et al. 2010). GenBank reference sequences, NG_008870.1 and NM_000553.4, were used for analyses. Western blotting analysis was performed as described, with minor modifications (Huang et al. 2006; Friedrich et al. 2010). Twenty-five micrograms of total protein was separated on NuPAGE 4–12% Bis-Tris gel (Invitrogen, Carlsbad, CA) and transferred to nylon membranes. WRN protein was detected with a mouse monoclonal antibody against aa 1074–1432 of human WRN protein (W0393, clone 195C, 1:2000 dilution; Sigma Aldrich, St. Louis, MO) and a biotinylated anti-mouse antibody (BA9200, 1:500 dilution; Vector Laboratories, Burlingame, CA). The reactions were visualized with Western Lightening Chemiluminescence Reagent (NEL100, Perkin Elmer, Waltham, MA) according to the manufacturer's instructions.

### Array comparative genomic hybridization analysis

Genome-Wide Human SNP Array 6.0. (Affymetrix, Inc., Santa Clara, CA) was used to exclude the possibility of genomic rearrangements including deletions and duplications and to map putative homozygous regions. Data handling, evaluation, and statistical analysis have been described in detail previously (Friedrich et al. 2010).

## Results

### Case reports

**Figure 1:**
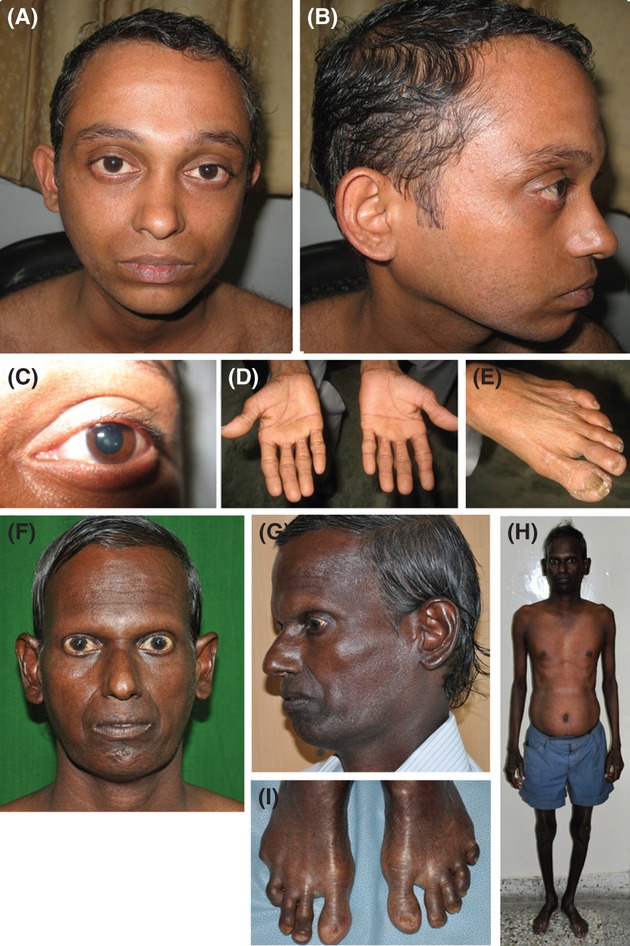
Profiles of Registry# KERA1010 (A–E) and VELO1010 (F–I). Photos of eyes (A, B, C, F, G), hands (D), and feet (E, I) show the presence of ocular cataracts, palmar keratosis, and overall aged appearance. (H) Thin limbs and central obesity.

**Figure 2:**
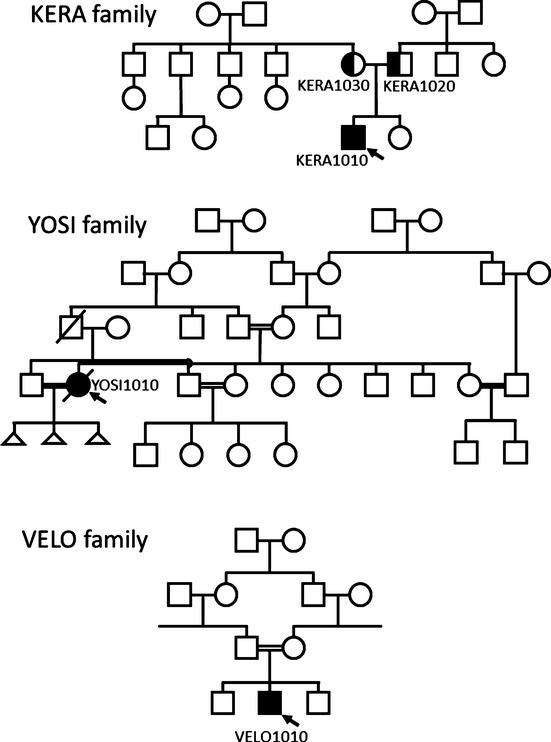
Pedigrees of three families with Werner syndrome. Filled-in symbols indicate affected individuals. Half filled-in symbols indicate individuals with confirmed heterozygous changes. Those with Registry# are individuals whose samples are sent to our Registry.

Registry# KERA1010 was a 23-year-old Indian man who was born in the state of Kerala to nonconsanguineous parents (Fig. 1A–E). His birth weight was 2.5 kg and his growth and development were normal until the age of 8–10 years, when he failed to undergo a pubertal growth spurt, and developed gynecomastia. His endocrine evaluation at that time was normal. He was diagnosed with bilateral posterior capsular cataracts and open angle glaucoma at 18 years of age (Fig. 1C). He underwent surgery for a left knee contracture at age 18. Examination at 23 years old revealed a short-statured man with normal cognition. His height was 148 cm (*Z* score −2.9 for Indian males in the Kerala state) (Mamidi et al. 2011), and weight was 29 kg (body mass index, BMI 13.2). His head circumference was 51 cm. He had scanty and prematurely gray scalp hair and facial hair, palmoplantar hyperkeratosis, thin limbs, and small hands and feet with a left single transverse palmar crease (Fig. 1D and E). His voice was hoarse, and he had bilateral vocal cord atrophy suggestive of presbylarynx. Endocrine evaluation had shown testicular atrophy (testicular volume of 12–15 mL bilaterally). There was no history of hypertension. Test for thyroid disease was negative. His lipid panel was normal except for elevated triglycerides (305 mg/dL; normal range 50–190 mg/dL) and cholesterol 235 mg/dL; normal 150–250 mg/dL). An abdominal ultrasound showed a diffuse fatty liver. Brain magnetic resonance imaging (MRI) and bone age were all normal. The X-ray study of the ankles did not show any sign of Achilles tendon calcification. Subsequently, he was diagnosed with type 2 diabetes mellitus at the age of 26 years and is currently controlled with pioglitazone and metformin. Family members were in apparent good health and included a 22-year-old sister, a 48-year-old mother, a 55-year-old father, four maternal uncles and one paternal uncle, and aunt and many first cousins, all of whom were unaffected (Fig. 2, top panel).

Registry# YOSI1010 was a 32-year-old British woman of Pakistani ancestry. Her parents were first cousins. A physical examination revealed short stature, pinched facial features, thin, prematurely gray hair, and dry-freckled skin. She was married to her first cousin and had had three miscarriages. Additional clinical features included type 2 diabetes mellitus, osteoporosis, and osteomalacia. She died at age 32 from a stage IV malignant melanoma of the right nasal cavity. She had four sisters, one of which was reported to have features of WS. There were two apparently normal brothers (Fig. 2, middle panel). A sample of stored DNA was received about a year after her death for *WRN* genetic testing.

Registry# VELO1010 was a 40-year-old Indian man born in the state of Tamil Nadu in India (Fig. 1F–I). His parents were first cousins. He was short (158 cm, *Z* score −1.08 for Indian males in the Tamil Nadu state) (Mamidi et al. 2011) and weighed 35 kg (BMI 14.0). He had pinched facial features (Fig. 1F and G), graying and thinning hair, including sparse pubic hair. He was diagnosed with bilateral cataracts at the age of 27, and underwent cataract surgery at the ages 37 and 39. Skin findings included palmoplantar hyperkeratosis, mottled pigmentation of the trunk, arms, and legs, and pseudoainhum of the right and the left of 5th toes which later led to autoamputation of the right of 5th toe. His voice was high pitched. The X-ray study showed specks of calcification at the site of insertion of Achilles tendon on the left leg. Radiograph of the forearm showed calcification at the site of insertion of triceps at the olecranon. Other findings included type 2 diabetes mellitus, muscle wasting, small testes, and inducible cardiac ischemia upon treadmill testing. He had no affected family members, was single, and had no children (Fig. 2, bottom panel).

### Identification of mutations at the *WRN* locus

**Figure 3:**
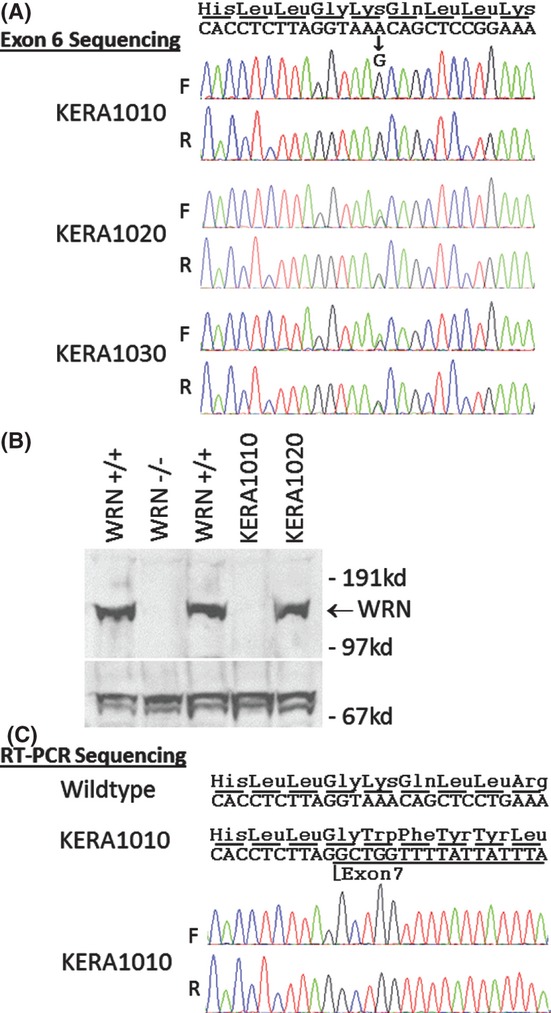
Sequencing analysis of KERA1010 pedigree. (A) Sequencing results of exon 6 detecting a homozygous c.561A>G change in KERA1010 and the same heterozygous change in the parents, KERA1020 and KERA1030. F and R indicate forward and reverse sequencings, respectively. (B) Western analysis of WRN proteins. *WRN*+/+ and *WRN*−/− are control individuals with known *WRN* genotypes. Nonspecific bands are also shown to indicate equal loadings. (C) RT-PCR sequencing of this region showed the skipping of the part of exon 6 starting 5 bp upstream of the mutated site. The wild-type sequence is shown on top. Altered sequence observed in KERA1010 is shown with underline. GenBank accession number NM_000553.4 was used as a reference.

The coding exons of the *WRN* gene were sequenced in all three index cases. Genomic sequencing of KERA1010 did not show any known *WRN* mutations or nucleotide changes consistent with null mutations, such as stop codon or frame shifts. Instead, this patient carried a homozygous c.561A>G silent substitution of unknown significance in exon 6 (Fig. 3A). The parents of KERA1010, KERA1020 (father) and KERA1030 (mother), both showed the expected heterozygous c.561A>G changes (Fig. 3A). The phenotypes of both parents were reported to be normal. This change was not listed in publically available single nucleotide polymorphism (SNP) databases. However, Western analysis of KERA1010 LCLs showed an absence of WRN protein, confirming the clinical diagnosis of WS (Fig. 3B).

As c.561A>G does not alter the corresponding amino acid (p.Lys187Lys), we investigated the possibility of an effect on mRNA splicing. The splicing prediction program at the Berkeley Drosophila Genome Project (available at http://www.fruitfly.org/seq_tools/splice.html) predicted that this change would create a new spice donor site 3 bp upstream of the putative mutation (ctcttagGTaa**G**cag where GT is a splice junction and bold **G** is the change observed in this patient). The usage of this new splice donor site would result in a 98 bp deletion of the 3′ region of exon 6, followed by a frameshift and chain termination after 13 amino acids (r.557_654del98, p.Lys187Trpfs*13). This mutant mRNA was expected to undergo non-sense mediated decay. The score for this putative cryptic splice site was 1.00. The score for a potential splice site in this program ranges between 0 and 1, where 1 indicates the most efficient splice site. The score for this putative cryptic splice site with the wild-type sequence (ctcttagGTaaacag) was 0.86. The score for the normal exon 6 splice donor site (tgcttatGTacgtgc) was 0.90. This indicates that the novel cryptic splice site created by c.561A>G would function as a more efficient splice donor site than the natural splice site of exon 6.

RT-PCR of *WRN* mRNA was performed in overlapping fragments because of the previously described relatively large size of its mRNA (Oshima et al. 1996; Friedrich et al. 2010). We sequenced five different RT-PCR fragments that covered exons 6–7. Four of the five RT-PCR products showed a normal *WRN* sequence without any deletions. One fragment revealed a 98 bp deletion at the 3′ end of exon 6 (Fig. 3C).

**Figure 4:**
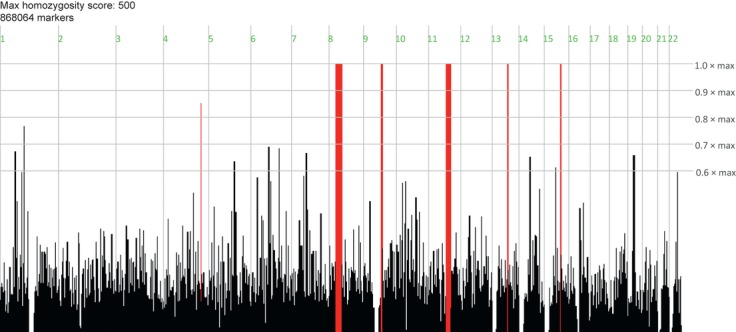
Homozygosity mapping of KERA1010. Homozygosity scores calculated with a genetic analysis software, ALLEGRO (http://www.decode.com/software/) are given along the *y*-axis relative to chromosomes on the *x*-axis. Chromosomes are concatenated from p-ter to q-ter from left to right. Regions of homozygosity are marked red.

Simultaneously, by the Genome-Wide Human SNP array, we excluded copy number variations in the *WRN* locus and identified six extended genomic regions of homozygosity with a maximum reachable LOD scores (Fig. 4). The *WRN* gene was located in the largest homozygous region, a finding which further supported the pathogenicity of the identified mutation.

Genomic sequencing of Registry# YOSI1010 showed the same homozygous mutation, c.561A>G. Due to the lack of available cell materials, RT-PCR or Western analysis of WRN protein could not be performed in this case. The genotypes of the *WRN* SNPs in Registry# YOSI1010 were the same as those detected in KERA1010 (Table 1), suggesting that the patients share the same ancestral mutation.

**Table 1:** Haplotypes of Indian and Pakistani Werner syndrome patients

Registry#:				YOSI1010	KERA1010	VELO1010
Origin:				U.K.	Kerala	Vellore
WRN mutation:				Exon 6	Exon 6	Exon 24
Exon	SNP	SNP ID	MAF			
2	c.96+26T>C	rs2737316	0.053	✓	✓	✓
6	c.513C>T,p.Cys171	rs1800389	0.427	✓	✓	
9	c.1161G>A,p.Met387Ile	rs1800391	0.061	✓	✓	
10	c.1350+22T>C	rs2737325	0.497	✓	✓	
18	c.1982-11delT	rs3087419	0.014	✓	✓	✓
21	c.2449-63A>G	rs4987034	0.232			✓
22	c.2631-67G>T	rs3087417	0.496			✓
25	c.3138+6C>T	rs3024239	0.467	✓	✓	✓
29	c.3384-126T>C	rs17652261	0.263			✓
29	c.3384-104T>A	rs17652297	0.364			✓
30	c.3572+24_3572+25insC	rs3087415	0.044	✓	✓	✓
34	c.4083C>T,p.Ser1361	rs1801196	0.383	✓	✓	
34	c.4099T>C,p.Cys1367Arg	rs1346044	0.278			✓

SNP, single nucleotide polymorphism; MAF, minor allele frequency. Nucleotide notations are based on the reference sequence, GenBank accession number NM_000553.4.

A novel stop codon mutation, c.2855C>A, p.Ser952*, in Registry# VELO1010 was previously listed in our mutation update (Friedrich et al. 2010). This mutation was not previously seen in over 130 previous *WRN* mutant cases. RT-PCR and Western analysis were not performed because attempts to establish LCLs failed due to the long time period between the blood draw and the arrival of the sample to our laboratory. The haplotype of the *WRN* alleles in VELO1010 was completely different from that of KERA1010 and YOSI1010. Table 1 shows the genotype of known SNPs detected in our *WRN* sequencing that are concordant between two c.561C>A mutants and discordant between c.561C>A mutants (KERA1010, YOSI1010) and c.2855C>A (VELO1010). These findings raise the possibility that there are at least two origins of *WRN* mutations in Indian/Pakistani patients.

## Discussion

We report on three pedigrees of WS of Indian/Pakistani origin. The clinical findings of the affected patients meet the clinical criteria for a definite (VELO1010) or probable (KERA1010 and YOSI1010) diagnosis of WS (Nakura et al. 1994; Yu et al. 1996; Oshima et al. 2012). The first patient from Kerala and the second patient, originally from Pakistan, shared the same mutation and same haplotype, which was distinct from the third case from Tamil Nadu. Geographically and culturally, Kerala and Pakistan are distant at the present time, yet given historically relevant patterns of migration, it is not inconceivable that individuals in the two populations could share a common ancestor.

The analysis of c.561C>A was complicated by the fact that this mutation did not destroy any of the normal splice sites of exon 6, allowing production of a very low amount of normally spliced mRNA. The amount of normally spliced mRNA in KERA1010 LCLs was not enough to produce WRN protein detectable by conventional Western analysis but was enough to be detected by standard RT-PCR. By contrast, the mutant mRNA with the 98 bp deletion underwent rapid non-sense mediated decay. This seems to be a satisfactory explanation as to why only one of five RT-PCR products sequenced in this study contained the 98 bp deletion. Genome-wide homozygosity mapping provided further evidence for its pathogenicity, whereas a possibility of genomic rearrangement was excluded by array comparative genomic hybridization (CGH). With the results of in silico analysis, we are confident that a splicing abnormality is the mechanism of this mutation.

We have previously reported on a series of ethnically specific *WRN* mutations that were seen in two or more independent pedigrees but not in patients with other ethnicities. These include Japanese, Sardinian, Turkish, Moroccan, and Dutch specific mutations (Satoh et al. 1999; Huang et al. 2006; Masala et al. 2007; Friedrich et al. 2010). Among them, the c.3139-1G>C mutation resulting in the skipping of exon 26 is widespread in Japan and regarded as a founder mutation (Goto et al. 1997; Matsumoto et al. 1997; Satoh et al. 1999). Similarly, the c.2089-3025A>G mutation resulting in insertion of a new exon (r.2088_2089ins106) is regarded as a founder mutation in Sardinian Werner patients (Masala et al. 2007). The prevalence of WS in these two populations is estimated to be as high as 1/20,000. Contributing factors to this high prevalence include the high rate of consanguineous marriage, heightened awareness of the disorder, and the fact that WS patients are able to live beyond reproductive ages, albeit with reduced fertility. The heterozygote frequencies of *WRN* mutations in India and Pakistan are not known. Given the high frequency of consanguineous marriage in India and Pakistan, it is conceivable that WS may be more common than currently recognized, and that with increased awareness, a founder effect in the India/Pakistan populations may be confirmed.
